# COMOKIT v2: A multi-scale approach to modeling and simulating epidemic control policies

**DOI:** 10.1371/journal.pone.0299626

**Published:** 2024-03-22

**Authors:** Patrick Taillandier, Kevin Chapuis, Benoit Gaudou, Arthur Brugière, Alexis Drogoul

**Affiliations:** 1 UMI 209 UMMISCO, IRD/Sorbonne University, Bondy, France; 2 LMI ACROSS, Thuyloi University, Hanoi, Vietnam; 3 UR MIAT, INRAE, University of Toulouse, Castanet-Tolosan, France; 4 UMR 228 ESPACE-DEV, IRD, University of Montpellier, Montpellier, France; 5 UMR 5505, IRIT, University Toulouse Capitole, Toulouse, France; University of Hong Kong, HONG KONG

## Abstract

The COVID-19 crisis demonstrated the importance of using models to understand, predict, and manage epidemics, in particular by assessing in advance the effect of different intervention policies. Numerous models have been proposed to answer a wide range of questions, from the impact of open borders to the effectiveness of neighborhood containment to the role of building ventilation in virus dispersion. However, the vast majority of these models are only suited to a scale of representation, analysis, or experimentation. In this article, we present the latest version of the COMOKIT toolbox, which is based on the integration of 3 models (COMOKIT-micro, COMOKIT-meso, and COMOKIT-macro) enabling these questions to be addressed at different geographical scales of analysis and exploration, from the building scale to the scale of entire countries. An application of these 3 models to various questions concerning public health policies against COVID-19 is presented and discussed.

## 1 Introduction

At the end of January 2020, in response to the COVID-19 pandemic, public health authorities around the world had to experiment with various combinations of interventions at different scales in a short space of time. However, as the pandemic progressed, the need for tools and methodologies to rapidly analyze the impact of these interventions and answer concrete questions about their effectiveness, scope, and timing became increasingly apparent.

COMOKIT, the COVID-19 modeling “kit”, was originally conceived as one such tool. COMOKIT is a combination of computer models, developed with the GAMA platform [1], which enables the impacts of different intervention strategies, as well as their combinations, to be explored *in silico* before their implementation. The first version of COMOKIT [2], designed as part of a Franco-Vietnamese partnership with funding from ANRS-MIE, focused on the study of interventions at the scale of a small town or neighborhood of up to ten thousand inhabitants, which at the time corresponded to the scale of policies implemented by the Vietnamese authorities. However, as the epidemic progressed, it soon became clear that this scale was not necessarily suited to all countries, or to the evolution of their public health policies. For example, while studying the effects of confinement or wearing a mask may make sense on a local scale, the same is not necessarily true for vaccination. Conversely, the success of a policy on a local scale often requires the ability to scale up in order to consider its adoption. In order to address more questions, or the same questions at different scales, the basic COMOKIT model was then transposed to address both very local issues at the scale of buildings (changes in people’s movements, reorganization of spaces to minimize propagation risks) and global phenomena at the scale of a large city or country.

To this end, version 2 of COMOKIT now offers 3 basic models built around the same common epidemiological model: the COMOKIT-micro model, which enables the movements and interactions of people in a building or group of buildings to be represented at a fine scale; the COMOKIT-meso model, which corresponds to the model integrated in the first version of COMOKIT, where individuals are still represented but in less details; and finally, the COMOKIT-macro model, which explicitly simulates groups of individuals rather than each individual, thus no longer being limited by the size of the population.

Still, with the aim of broadening the questions COMOKIT can help answer, significant work has been carried out to generalize the platform to other infectious diseases, and to enable simplified representation and management of variants and vaccination policies. COMOKIT v2 can now take into account the fact that several variants of the same infectious agent can coexist and compete within the same human population. As far as vaccination is concerned, COMOKIT v2 builds on the explicit representation of intervention policies in COMOKIT v1 to integrate vaccination policies and their impacts, notably on the contamination process, taking into account acquired immunity, and the evolution of the disease in infected individuals, notably its severity.

This article is organized as follows. In section 2, we present a state-of-the-art review of agent-based models that have been designed or dedicated to the study of interventions against COVID-19, allowing us to point out the shortcomings of existing models (and in particular, their inability to generalize beyond a specific case study or scale). In section 3, we present the COMOKIT toolbox, the 3 models that make it up, as well as examples of applications of these models. The 4 section returns to the interest of considering different modeling and simulation scales within COMOKIT and how, thanks to the agent-based underpinning model they share, they can be effectively combined to overcome scaling problems in epidemiological modeling. Finally, section 5 concludes by listing some of the limitations of the current version of COMOKIT and presenting its prospects for evolution and application to different contexts.

## 2 Agent-based models to study the diffusion of COVID-19

The COVID-19 pandemic has led to the emergence of a new role for scientists, with stringent pressure on epidemiological simulation models that were supposed to *lead the response* to the pandemic [3]. In that context, computer models had been used earlier in the pandemic to assess how effective Non-Pharmaceutical Interventions (NPI) were, and which one should we endorse as the most effective. Among classical mathematical and other modeling approaches, ABM quickly appears as one of the most relevant techniques to test various intervention policies and assess effectiveness taking into account realistic social and spatial aspects of the COVID-19 spread. In this section, we consider a rapid review in the form of a synthesis of existing systematic reviews of ABM that have been used to study COVID-19 diffusion, interventions, and consequences.

To the best of our knowledge, [4] conducted the most systematic and reliable review of agent-based simulation models of COVID-19 transmission and mitigation processes. This review analyses 126 papers describing ABMs of COVID-19 as of June 2021, testifying that the ABM community has been very active in studying and modeling the pandemic. Its authors focus more precisely on the modeling of interventions, either pharmaceutical or non-pharmaceutical, to mitigate the transmission and propagation of the disease. Results show that the existing models are very heterogeneous in terms of objective, number of individuals simulated, way of modeling transmission dynamics, disease states, human behaviors, and interventions. For instance, three of the most influential ABMs do not explicitly model agent interactions: Ferguson and colleagues [5] represent agents as records within geographical cells, with a probability of contact depending on a kernel function; while OpenABM-COVID19 [6] and Covasim [7] rely on a fixed multi-layer contact network. The article also shows that there is a lack of transparency in the models presented, which often do not describe the data used, limiting their use for decision support. Lastly, none of the 126 models examined provides a multi-scale representation of COVID-19 propagation, even though the spatial scale ranges from a small neighborhood of a few hundred agents to an entire country.

Recently, another analysis of the contribution of ABM to COVID-19 modeling was published by [8]. Although not a systematic review, it helps to highlight the shift in focus of crisis response models towards the wider post-crisis context. For example, many models focus on the resilience of socio-economic systems in the face of a pandemic. This underlines ABM’s ability to assess the impact of mitigation policies on epidemiological aspects such as disease spread, healthcare, or immunity, but also the consequences of interventions on the socio-economic context, such as increased poverty [9], business closures [10] or welfare [11].

A more recent review, with a broader scope, has been published in [12]. In addition to ABM, the authors consider discrete-event simulation models and system dynamics in their review, although the cumulative number of articles for the latter two is more than three times less than for ABM alone. They propose a classification of articles into four categories: those focusing on transmission, propagation prediction, intervention evaluation, and cross-sector impact assessment. Only the last category is not dominated by ABM approaches. As far as transmission is concerned, ABM brings interesting features in addition to classical epidemiological modeling: transmission in a particular location (e.g., a school, a hospital, a church, and even the ship Diamond Princess) and flexibility in terms of determinants (e.g., natural disasters or dissemination of misinformation) and outcomes (e.g., economic losses or online panic). When it comes to predicting the spread of COVID-19, while targeting classic indices such as infections, mortality, or demand for intensive care units, ABM enables spatially explicit records to be taken into account in different scenarios involving NPIs. Evaluation of intervention against COVID-19, both PI and NPI, is the main task for which ABM has been used (54% of all articles reviewed), 3/4 at a regional or national level. Finally, a small number of ABMs focus on assessing the impact of COVID-19 on economic or social sectors and healthcare. An interesting trend was highlighted by the authors concerning the need for simulation models capable of answering counterfactual hypothetical questions in order to implement prevention and control measures at an organizational level. The authors concluded the study by mentioning the substantial benefits of developing more hybrid methods and models to reduce computational load and provide more appropriate and rapid support at micro/macro decision scales.

Similarly, [13] proposed a bibliometric review of simulation models mixing ABM with discrete event models and system dynamics. The result of the author’s synthesis classified models into five research streams, from economic modeling to organizational and political modeling, via conceptual and statistical modeling. These classes emerge from the analysis of global co-occurrences and highlight the diversity of ABM narratives applied to the study of the spread, mitigation, and outcomes of COVID-19. In terms of the significance of each type of class, economic models (the smallest group accounting for 10% of contributions studied) focus on assessing and mitigating socio-economic side-effects due to intervention and disease spread, statistical modeling (±11%) concentrates on predicting and simulating the correspondence of results with real data, while the other three classes emphasize the need to provide solutions to a whole range of problems, from decision-making through what-if scenarios for conceptual modeling (31%), to localized and specialized risk assessment in buildings for organizational modeling (22%), to the evaluation, implementation and monitoring of real-world interventions to support policy-makers in policy-based modeling (26%).

Overall, studies on ABM highlight the diversity of this new field of research. This diversity can also be seen in the so-called hybrid approaches, combining ABM with micro-simulation, individual-based modeling, system dynamics, or discrete-event simulations. We can note a wide variety of choices in the representation of interactions—through networks, activities, or via a probabilistic approach—interventions—from very simplistic scenarios (e.g. reduced contact probability) to realistic spatially explicit activity restrictions and social distancing—and finally infection—from a simple SIR model to complex transmission processes relying on dynamic individual viral loads. It is also possible to see that modeling objectives are very diverse, from studying the consequences of mitigation measures to testing real interventions in very specific contexts. It should be noted that none of the models examined has demonstrated its ability to represent the propagation of COVID-19 at several spatial scales (e.g. from building to city). In the following sections, we show how COMOKIT v2 provides this capability, which is a key modeling factor for better understanding the COVID-19 pandemic and preparing for possible future outbreaks.

## 3 COMOKIT v2

### 3.1 General presentation of COMOKIT

COMOKIT v2 proposes 3 models (COMOKIT-micro, COMOKIT-meso, and COMOKIT-macro) that share common elements. More precisely, COMOKIT is composed of a core that includes a set of generic and abstract agent species and 3 models that reuse these abstractions. the COMOKIT-meso model corresponds to COMOKIT v1 [2].

**Fig 1:**
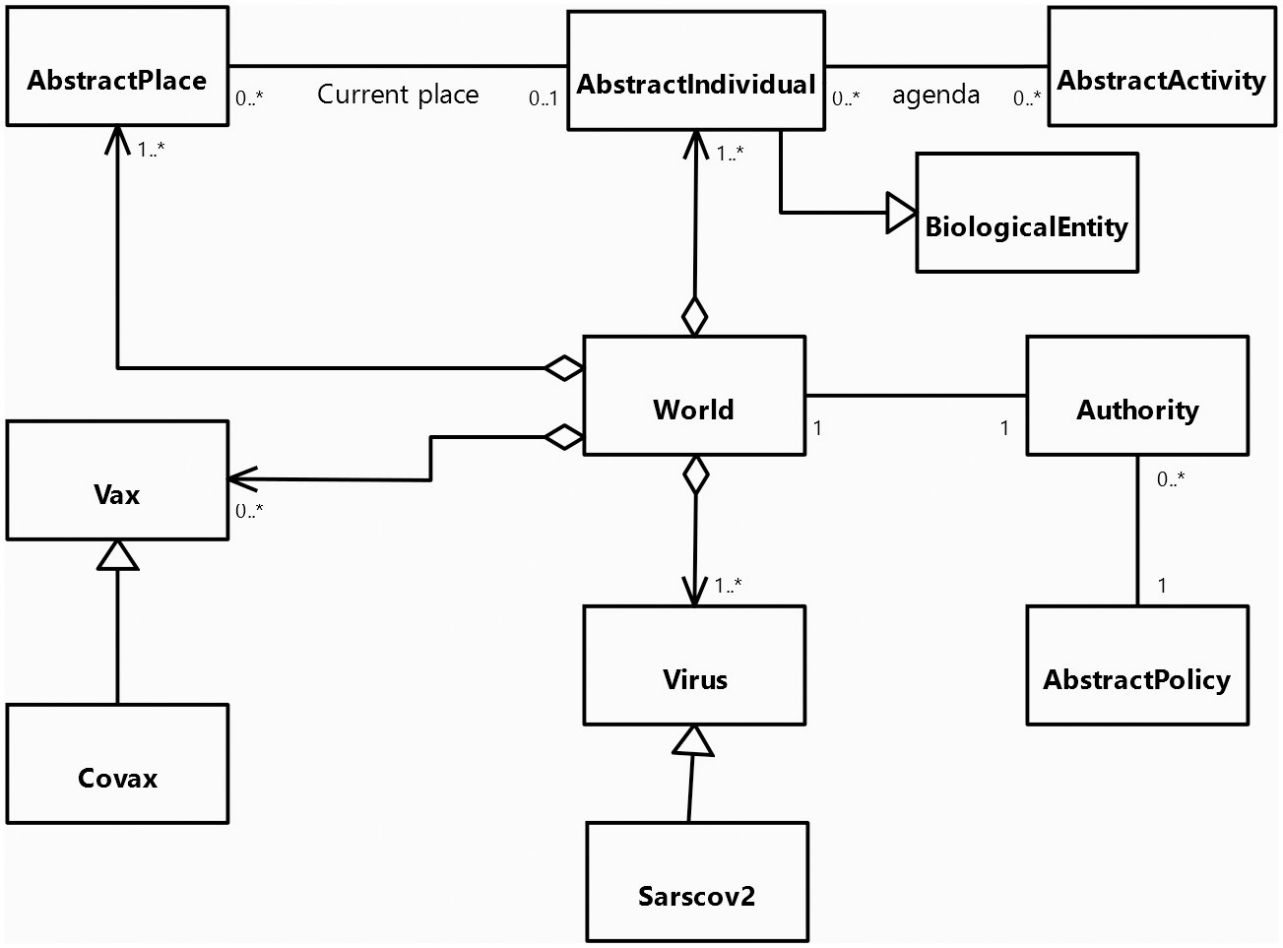
UML class diagram of the COMOKIT core entities.

Fig 1 presents the UML class diagram of the core of COMOKIT. The main entity of the model is the *AsbtractIndividual* agents: it represents individual inhabitants of the world with their individual characteristics (age, sex, employment status) and their epidemiological status, like epidemiological individual-dependent values (e.g. latent time, infectious time) that for the most part are inherited from *BiologicalEntity*. On the epidemiological side, the model features a Virus entity which makes it possible to represent variants from the source strain of COVID-19. Each infection is caused by a particular *Virus* entity, with *BiologicalEntity* having an immunological response based on past infection history and vaccination. Regarding social aspects, *AbstractIndividual* agents perform their daily activities (including going to work, school, shopping, eating outside…) depending on their personal agenda. This agenda is a generated set of *AbstractActivity* linking each activity to a current time (day and time). *AsbtractIndividual* agents are located in an *AbstractPlace* where they carry out their *AbstractActivity*.

As our main goal is to simulate and compare the application of various mitigation and control policies, a specific focus is made on policies that modify the population behavior to reduce contacts and thus infection between people. As a consequence, the performance of an Individual agent’s *AbstractActivity* is constrained by the allowance of the *Authority* agent. This agent manages the various *AbstractPolicy* that are adopted. When an *AsbtractIndividual* agent asks it for authorization to perform a given *AbstractActivity*, the *Authority* asks all the policies it has adopted whether any of them denies the given *AsbtractIndividual* to do the given *AbstractActivity*. Examples of *AbstractPolicy* include total containment, closing the schools, closing the workplaces… These policies can be limited to a given area (using *SpatialPolicy*) or be more or less tolerant (e.g. containment can be for everybody or for everybody but some people, or for a fraction of the population, using *PartialPolicy*).

One particular *AbstractPolicy* concerns vaccination. In this context, COMOKIT integrates the notion of vaccine in the form of *Vax* agents. A *Vax* agent represents a particular type of vaccine with its own characteristics against a given *Virus*. Several vaccines for the same disease can coexist at the same time.

**Fig 2:**
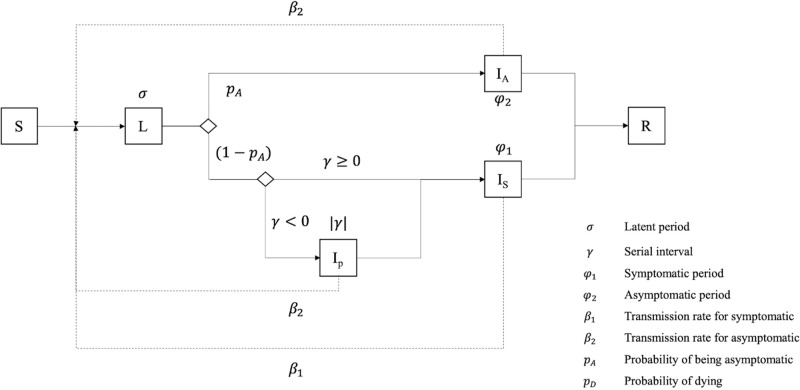
Epidemiological model of *BiologialEntity*.

Concerning the epidemiological sub-model, which is common to all 3 models (Micro, Meso, Macro), it is partly based on the SEIR model using a final state machine structure (Fig 2). We assumed the whole population starts the simulation in a Susceptible state (S) (as this is an emergent disease, nobody is immunized). When an Individual is in contact with an Infectious agent or located in an infected building, it can become infected and move to the Latent state (L), where individuals are not yet infectious, depending on the successful transmission rate and its own immunological response. The successful transmission rate here is defined as the probability for one contact at a given step to be infected by an Individual and a particular strain of COVID-19, which can be altered by the infectiousness of the transmitted virus and the immune response (based on history of infections and vaccination) of the recipient *BiologicalEntity*.

Once the latent period has expired, the Individual agent will move to one of the three infectious states: asymptomatic (Ia), presymptomatic (Ip), or symptomatic (Is). The transmission risk of a susceptible individual will depend on being symptomatic or asymptomatic and will be multiplied by a viral factor which may be different for each Individual and COVID-19 virus strains. We consider that asymptomatic and presymptomatic Individuals share the same transmission rate.

**Fig 3:**
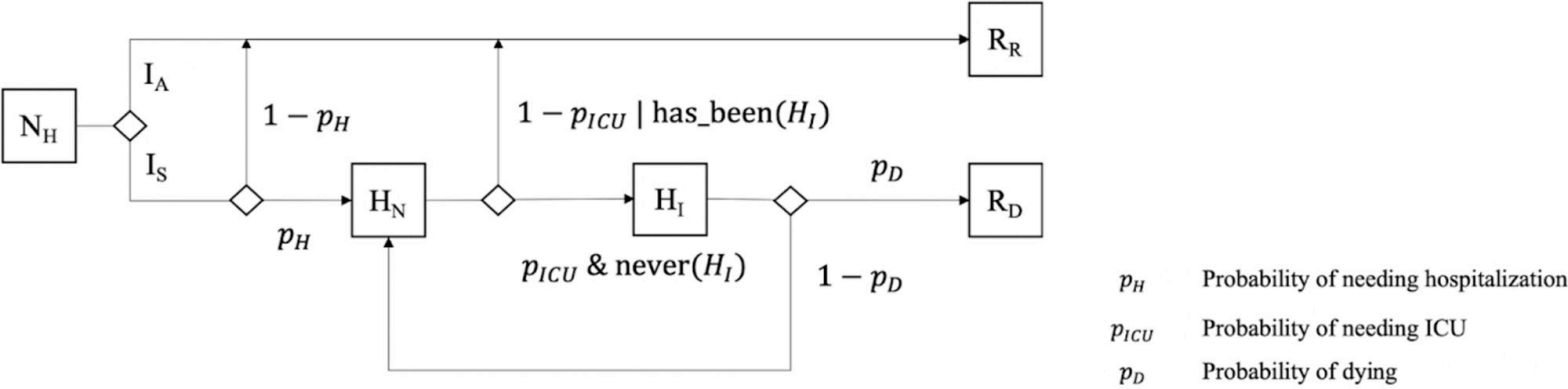
Hospitalisation model of *BiologialEntity*.

Once the infectious period is over, Individual agents reach the Removed (R) state, representing all individuals that have been infected, but are not infectious anymore. To represent deaths and recoveries, we decided to represent the current clinical status of the Individual agent. Symptomatic Individuals begin with a clinical status set to not needing hospitalization (NH) (Fig 3). Individuals in the asymptomatic, latent, or presymptomatic states will not need hospitalization. However, symptomatic Individuals have a probability of needing to be hospitalised (and thus move to the clinical status needing hospitalisation HN). Once an agent is in state HN, it has a probability to require being admitted to an Intensive Care Unit (ICU), thus moving to the clinical status HI. (otherwise, it will be considered as recovered (RR) after the infectious period). If the Individual agent is not taken to a hospital before the end of its expected period needing ICU, it will be considered dead due to lack of treatment (corresponding to the clinical status dead RD). For Individuals in ICU, the hospital will decide on the clinical status according to the probability of dying in ICU cases. Agents that do not die during their ICU stay, are considered recovered once they do not show symptoms anymore (i.e. not in the state symptomatic) and are tested negative for x consecutive days. On the contrary, agents that do not need ICU will be automatically considered as recovered (RR) after the infectious period.

We describe in the following sections the 3 models that will reuse some or all of the abstractions defined in the core as well as the epidemiological sub-model. For each model, an application example is provided. The source code of COMOKIT as well as the code and data of the example applications can be found on the GitHub page of COMOKIT, link up as of September 2023: https://github.com/COMOKIT.

### 3.2 COMOKIT Meso

#### 3.2.1 Overview

This model aims at simulating and comparing the application of COVID-19 spread mitigation policies at the scale of a closed commune, with the transmission of the disease being modeled at the individual scale. Its purpose is to support deciders and researchers in answering questions about containment strategy, mask-wearing impact, or vaccination policies. The simulations are executed at the scale of a small city or a district of a city (around 10,000 inhabitants). The smallest considered spatial units are individual buildings.

The simulations are not launched from a specific starting date, but rather from the introduction of the first infected cases in the population and will run until the end of the epidemic. The simulation step is set to 1 hour. As a consequence, given the limited size of the simulated area, movements from one activity place to another one are not simulated: individuals are always located in an activity place (that can be a close building or even an outdoor park). The underlying assumption is that no infection can occur during the move time.

#### 3.2.2 Entities

The Meso model includes all the abstractions of the core. It extends the notion of *AbstractIndividual* with the *Individual* agents which integrates additional information. Indeed, *Individual* agents’ attributes include their relatives (their family which corresponds in our model to the other *Individual* living in the same flat in a Building), their friends (with whom they can share activities), their colleagues (work colleagues or classmates) and their home, working place and school *Building*.

*Building* agents are spatial entities extending *AbstractPlace* where the *Individual* agents can perform an *Activity*, this *Activity*, that extends *AbstractActivity*, depending on the *Building* type. Two special *Building* types have been defined as they have an important role in the simulation: *Outside* and *Hospital*. The *Outside* agent represents all the buildings outside of the simulated area: it is used to represent the fact that people can be working or doing activities outside the area under consideration. For this zone, a particular contamination dynamic is applied. *Hospital* agents will be the place where, in some situations, sick *Individual* agents with critical symptoms can be contained and healed.

Regarding social aspects, *Individual* agents perform their daily activities (including going to work, school, shopping, and eating outside…) depending on their personal agenda. This agenda is a generated set of *Activity* that can be shared by several individuals (e.g. going to a restaurant with some friends), depending on the age and family status of the *Individual* agent. An *Activity* is mainly a way to choose the spatial unit(s) in which the *Individual* agents have to be located at each simulation step. The choice of the spatial unit to carry out an *Activity* depends on several factors: the first one is the preferences of the *Individual* that is defined according to his/her age and sex: for a leisure activity, a child may prefer to go to a game center while an older person may prefer to go to a movie theater. Once the type of spatial units is defined, the choice of the place among those of the chosen type depends on the modeler’s decision. COMOKIT offers 3 basic models: choice of a random place, choice of the closest place, or a gravity model. For the latter, the probability of choosing a place will depend on the area of the place and on its distance to the *Individual* agent (the larger the place and the smaller the distance to it, the greater the chances of choosing this place).

We have also defined additional specific *Activity* species to represent the main classical kinds of *Activity*: *visiting*_*neighbor*, *working*, *staying*_*home*, *studying*, *visiting*_*friend*. Of course, customs activities can also be created from the generic *Activity* species.

**Fig 4:**
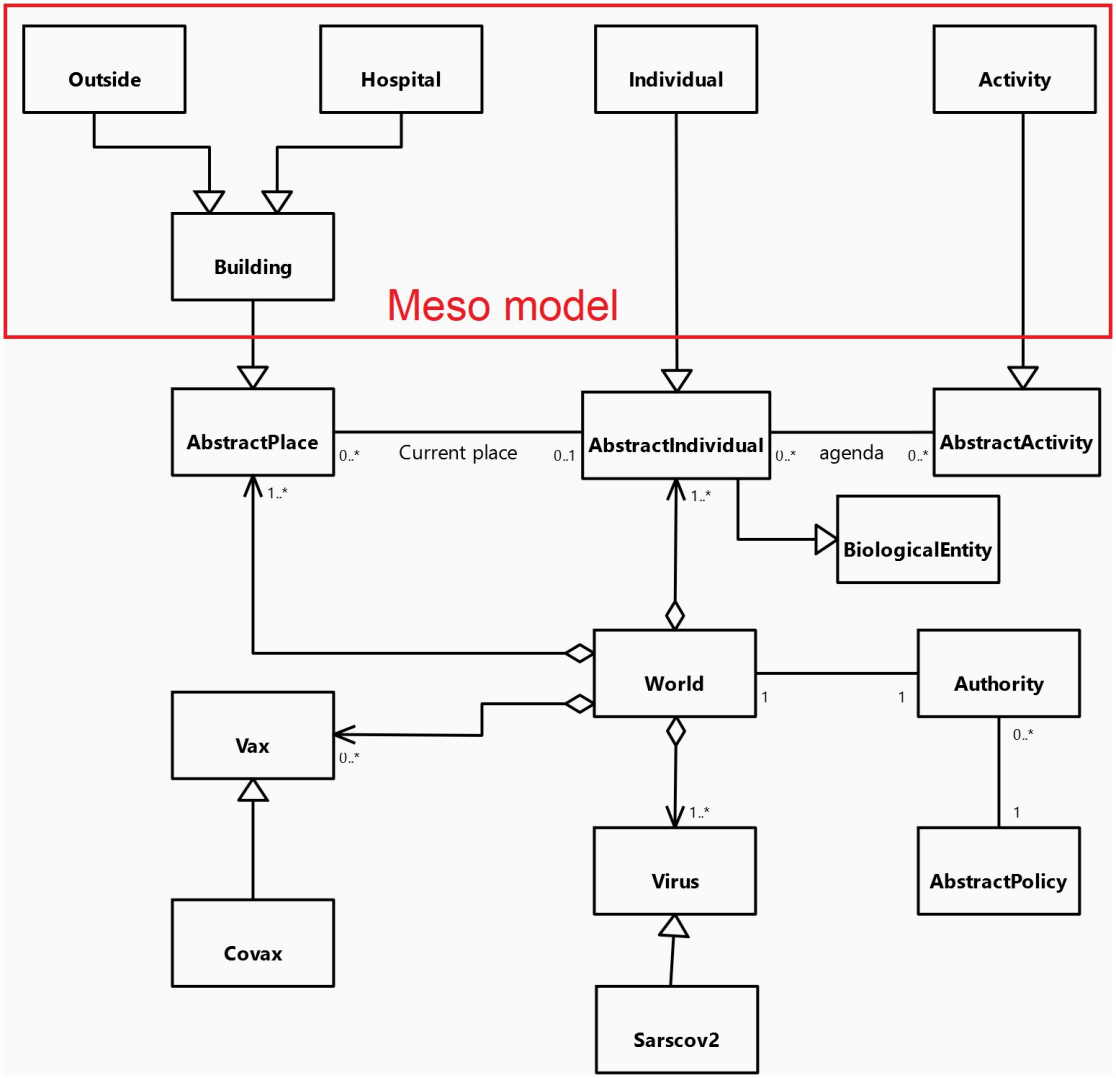
UML class diagram of the COMOKIT Meso entities.

All the agent species are summarized and organized on the UML class diagram presented in Fig 4.

#### 3.2.3 Processes

The model simulates the spread of COVID-19 in a population at the individual level under the control of mitigation policies or individual behaviors (such as wearing masks). The dynamics of the model can thus be summarized by three main dynamics: the epidemic dynamics, the hourly activities of the Individual agents, following their agenda to go from Building to Building, and the dynamics of policy adoption and application.

There are two different pathways of infection for *Individual* agents: either through Individual-to-Individual transmission or through the persistence of the virus in the environment. When an infectious *Individual* agent is located in a building, it can release a virus load inside the building, which can survive several hours. *Individual* agents who will come to this building can thus become infected by the viral load present in the building itself. As soon as an *Individual* agent is infected, its epidemic status will be described by a set of states and transitions (given probabilities taken from the up-to-date COVID-19 literature).

A simulation step starts with the evolution of the viral load in a building (it decreases over time, before disappearing). Then the *Individual* agents behave. They first evaluate whether they are infected or infect other *Individual* agents or the current building in which they are located. They then update their epidemic status and their individual behavior related to mask-wearing. Finally, they execute their daily activities: they find the activity corresponding to the current hour, ask the *Authority* agent whether they are allowed to execute it, and act in accordance.

Finally, the *Authority* agent checks its current *Policy* agents and tries to apply it (executing a test campaign for example).

#### 3.2.4 Example of application

**Fig 5:**
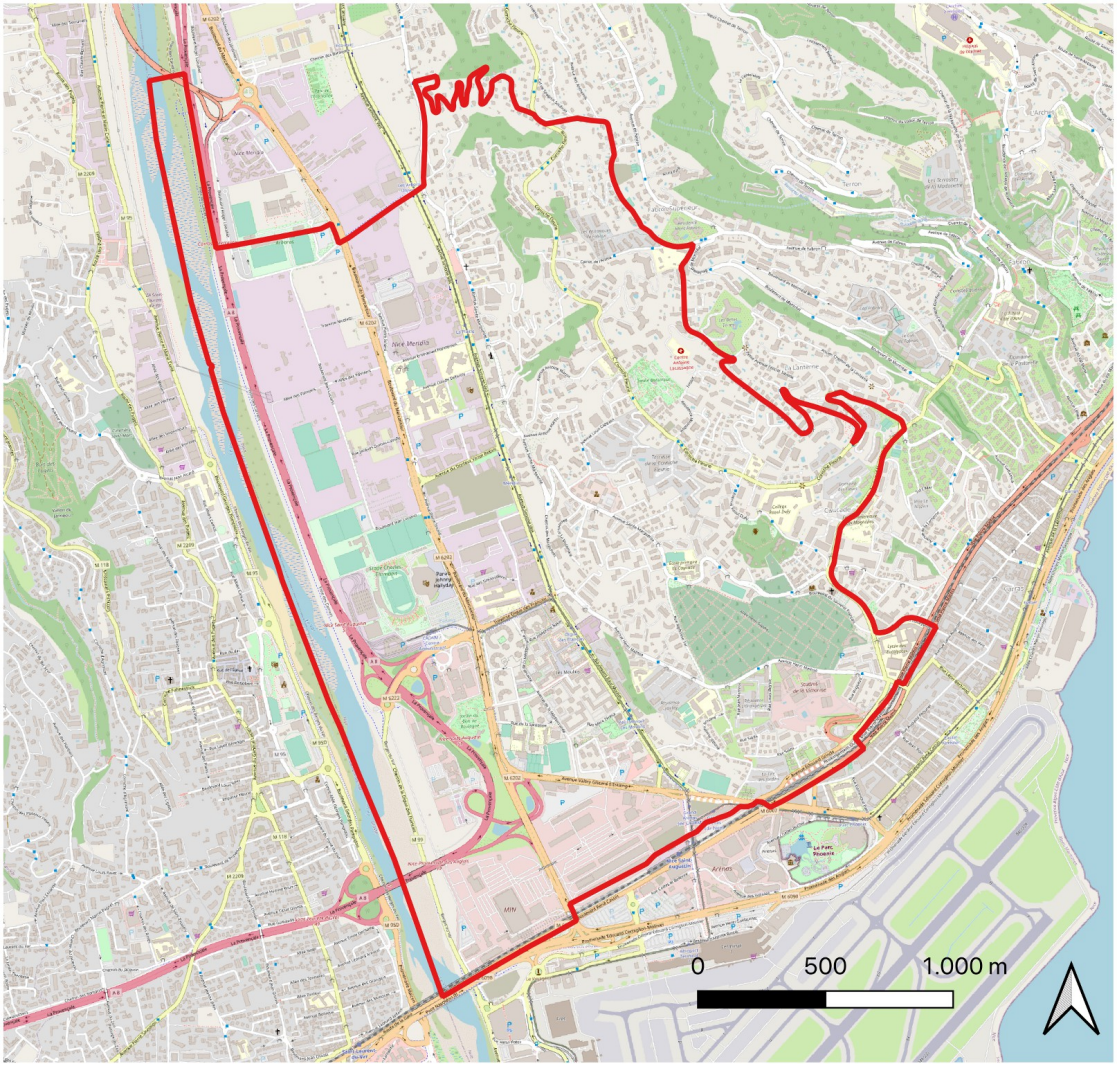
Map of the Nice district considered for the application of the Meso model (data source: OpenStreetMap).

In order to illustrate the use of the Meso model, we propose an application for the study of localized and dynamic containment policies in a suburban residential area of the city of Nice (France). This area is located outside the city of Nice and is characterized by a low population density in single-family homes and a low level of services. Functional specialization is the hallmark, which also includes some concentrations of car-based commercial buildings. Fig 5 shows the map of the area used to apply the Meso model. The area’s population is made up of 9500 individuals. To generate the population and their agenda, we used COMOKIT’s integrated generator.

For the studied policy, we consider a spatialized containment around identified cases: as soon as a case is identified by a test, the area around the dwelling place of this case (at a given distance) will be contained: people living in the isolated area will no longer be allowed to carry out any activity. The challenge is to see the impact of this policy, depending on the distance considered, on the number of infected persons, and on the number of activities that could not be performed.

As far as the testing policy is concerned, we consider that every day random tests are carried out on 0.5% of the population. These people are chosen at random, regardless of previous tests or their epidemiological status. For the spatialized containment policy of identified cases, we tested different distance values for containment implementation: 0*m* (just the building in which the test person lives), 50*m*, 100*m*, and 500*m*. We compare this policy with a reference scenario where no policy is applied.

An important element of this case study is that testing this policy requires taking into account individual people as it depends on the status of each (tested, infected) and their location. It also requires to take into account the location of the other individuals to know if they are in a containment area. Thus, it would not have been possible to use an aggregate model to test this type of policy, as the impact of the policy is individualized and localized.

In order to balance the model’s stochasticity, 50 simulations were carried out for each parameter set and results were averaged over the replicates. The number 50 comes from the stochasticity analysis carried out for this model in [2].

**Fig 6:**
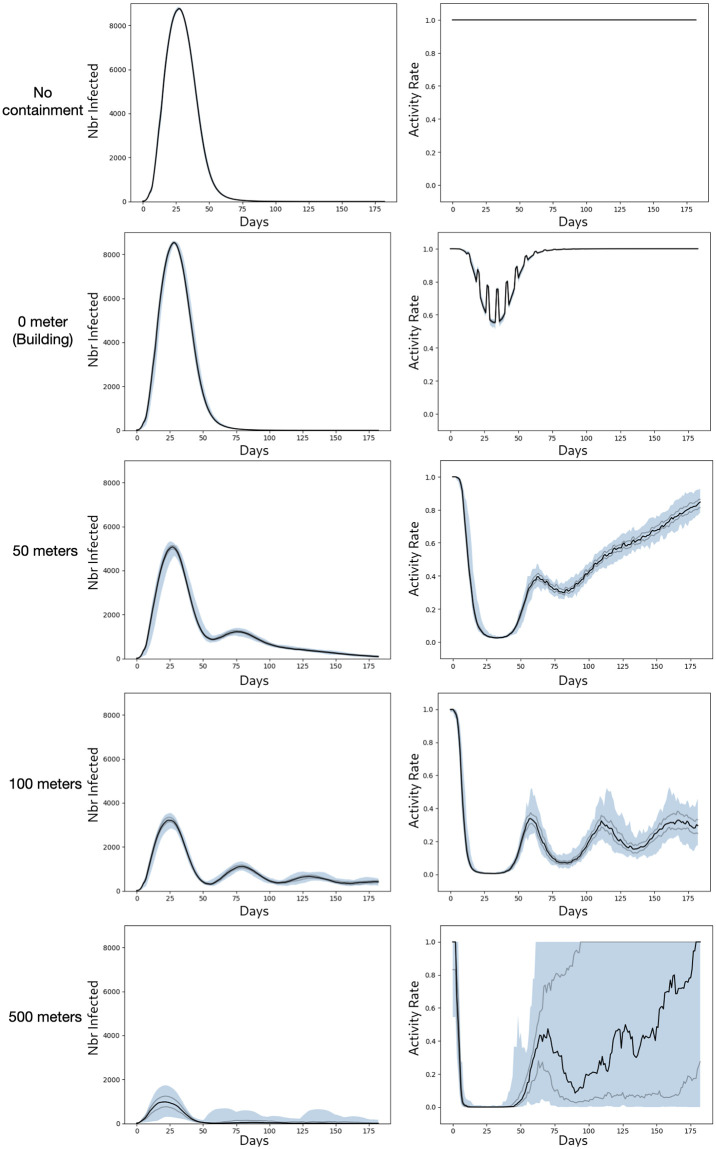
Meso model: Evolution of the number of infected people and of the rate of allowed activities. The black curve represents the median, the grey curves the first and 3rd quartile respectively, and the blue surface the difference between min and max.

Fig 6 shows the evolution of the number of infected people and the rate of people who were able to carry out their activity.

The results show that the policy implemented can indeed greatly help to reduce the risk of the disease spreading but with a potentially strong impact on people’s activities. While the impact of confinement to buildings (*O* meter) is limited both in terms of the spread of the disease (with only a very slight flattening of the infection curve) and in terms of people’s activities, as soon as confinement no longer concerns just the building, but also those in the neighborhood, the impact becomes much stronger: for a distance of 50 meters, the infection curve is really flattened, and at the peak of contamination, the rate of people no longer able to carry out their activities rises to over 80%. However, this rate diminishes as the number of cases decreases. A distance of 100 meters flattens the curve considerably, but an oscillating effect can be observed: as soon as the number of cases decreases, the number of people affected by the restrictions decreases, and the disease starts up again as few people are immune (i.e. have been contaminated before). Finally, in the case of a 500 meter confinement around detected cases, most of the time, the neighborhood will be totally locked, which means that the disease will not spread at all (median case). However, in some simulations, we may find that cases of contamination persist, blocking the entire population for a very long time.

In conclusion, this policy can be really effective, but in addition to being complex to implement, it can have very negative effects on people’s activities.

### 3.3 COMOKIT-micro

#### 3.3.1 Overview

This model aims at simulating the spread of COVID-19 at the scale of a building or a small set of buildings. Its purpose is to support deciders and researchers in answering questions about mask-wearing impact, ventilation of rooms, or vaccination policies. The simulations are executed at the scale of a set of buildings, with several floors. The smallest considered spatial units are individual rooms. The simulation step is set to 1 minute.

#### 3.3.2 Entities

Like in the COMOKIT-meso model, the core entity of the model is the individual type of agents, named *BuildingIndividual* that extends *AbstractIndividual*: it represents individual users of the buildings. The individual agent’s behavior is driven by their agenda attribute that associates to some date a *BuildingActivity*. A *BuildingActivity* is mainly a way to choose the *Room* and eventually the location in the room where the individual agent will have to move.

We have defined some specific *BuildingActivity* species to represent the main classical kinds of activities: *ActivityLeaveArea*, *ActivityGotoOffice*, *ActivityGotoRestaurant*, *ActivityGotoRoom*. Of course, custom activities can also be created from the generic *BuildingActivity* species.

In terms of spatial entities, *Room* agents are where the individual agents can perform an *BuildingActivity*. A *Room* has one or several *RoomEntry* and several *Wall*. It is located on a specific floor of a Building agent. A specific type of *Room* is *Elevator*, allowing Individual agents to pass from one floor to another one. In addition to *Room* agents, a *Building* agent has one or several *BuildingEntry*. The global environment is characterized by *Building* agents, but also by one or several *AreaEntry*, representing an exit from the studied area (an agent will have to go to this spatial entity to carry out activities outside the zone) and by *PedestrianPath*, representing the path that individual agents can follow to move. Among the spatial entities, we can make a distinction between the ones that can contain a viral load and thus allow environmental contamination (such as *Room* and *Elevator*); the virus management feature is factorized in the *AbstractPlace* species) and the other ones that cannot induce environmental contamination. Note that while *AreaEntry* inherits from *Room*, it has no associated viral dynamics: there is no virus load in it but is just considered a place where agents must pass to exit the study area.

Finally, the last type of spatial entity is the *UnitCell* agents, which allow to take into account the contamination through objects. More precisely, we don’t consider individual objects (table, door handle, etc.) but areas defined by *UnitCell* dividing *Room* agents into smaller subareas representing specific epidemiological contexts. Indeed, for each *UnitCell*, a viral load is defined, corresponding to the contamination of objects in that area.

**Fig 7:**
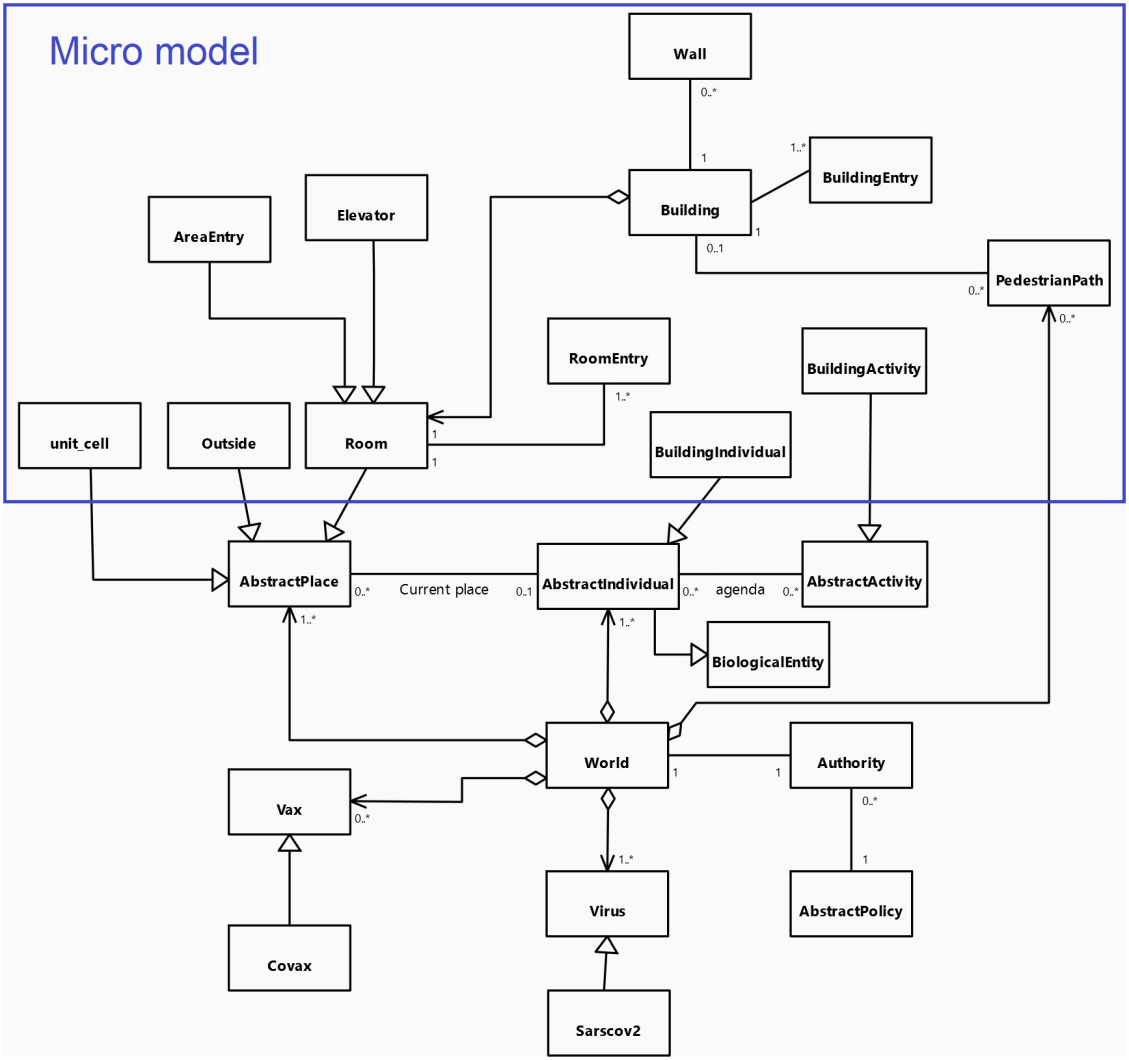
UML class diagram of the COMOKIT Micro entities.

All the agent species are summarized and organized on the UML class diagram presented in Fig 7.

#### 3.3.3 Processes

The dynamics of the COMOKIT-micro model is close to the one of the COMOKIT-macro model and can be summarized by two main dynamics: the epidemic dynamics and the activities of the *Individual* agents, following their agenda.

Contrary to the COMOKIT-meso model in which, the agents are just located in a building and “teleport” from one building to another according to their activity, the COMOKIT-micro model allows to represent the exact location of the agent in the building (defined in a space x,y in a room, which is itself on a precise floor of a building) as well as their displacements from room to room, or even from building to building. This fine consideration of the location of agents allows to represent much more finely the dynamics of infections. Thus, similarly to the model proposed by [14], three different pathways of infection for Individual agents are considered: either through Individual-to-Individual transmission, through the persistence of the virus in the air, or through the persistence of the virus on physical objects. When an infectious Individual is located in a building, it can release a virus load on the unit cell it is located in, to represent the persistence of the virus on physical objects. In addition, it can release a virus load in the room it is located in, to represent the persistence of the virus in the air. Individuals who will come to a unit cell and/or a room can thus become infected by the viral load present in the unit cell/room itself. Note that since a room is made up of unit cells, an individual located in a room can be infected both by the viral load of the room and of the cell unit. As soon as an Individual is infected, its epidemic status will be described by a set of states and transitions (same epidemiological model—defined in the Core—as for Meso).

The dynamic of the model is very similar to the one of the Meso model. A simulation step starts with the evolution of the viral load in the unit cells and in the rooms (or more generally all the spatial entities that can contain a viral load): it decreases over time, before disappearing. Then, the Individual agents evaluate whether they are infected or infect other Individuals, or release virus load in their current unit cell/room. They then update their epidemic status and their individual behavior related to mask-wearing. Finally, they execute their activities: they find the activity corresponding to the current time and act in accordance. The activities can consist of moving toward a specific room, or toward a specific place, but also of moving inside a specific room. Indeed, the model allows to take into account that some activities require frequent movements inside a room.

#### 3.3.4 Example of application

**Fig 8:**
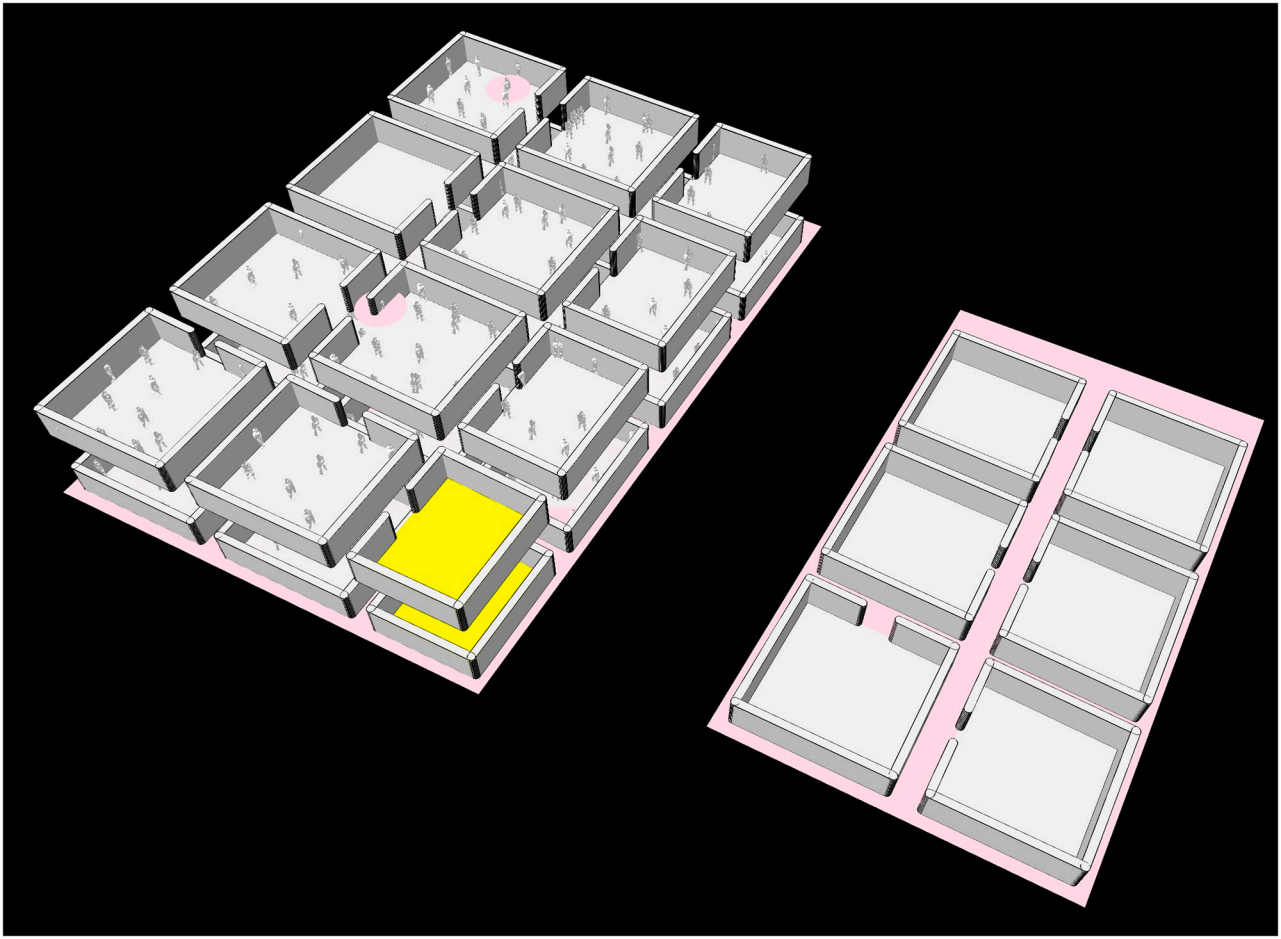
Representation of the area considered for the application of the Micro model.

We propose as an example of applications, a typical case of a complex composed of a main building serving as office (composed of 2 floors) and a second building intended for the restoration (composed of 1 floor). Fig 8 shows a snapshot of the simulation presenting the two buildings. We consider that 500 individuals work in this area. The population was created using the built-in generator of COMOKIT-micro that allows to create default workers: every worker has a certain probability of doing a night shift. If they work during the day, they will take a break at lunchtime to eat in the restoration place, and then return to their workstation before leaving in the evening. If the worker is on night duty, he/she won’t be able to go to the restaurant and will remain at his/her workstation all night.

Two types of policies are tested: the social distancing and the ventilation of rooms. Concerning this second policy, the idea is to ventilate the room in order to reduce the virus load in the room. In this experiment, we assume that all rooms have the same level of ventilation: either all are ventilated, or none are. Concerning the social distancing, as long as it is possible, people arriving in a new room will seek to be at a certain distance from those already installed. If this is not possible, then they will accept to be closer to the others.

This type of spatially explicit policy within buildings is not possible to consider directly in the COMOKIT-meso model as the transmission process is done regardless of individual locations within buildings (which would require too much computational time).

To take account of the model’s stochasticity, 50 simulations were carried out for each parameter set.

**Fig 9:**
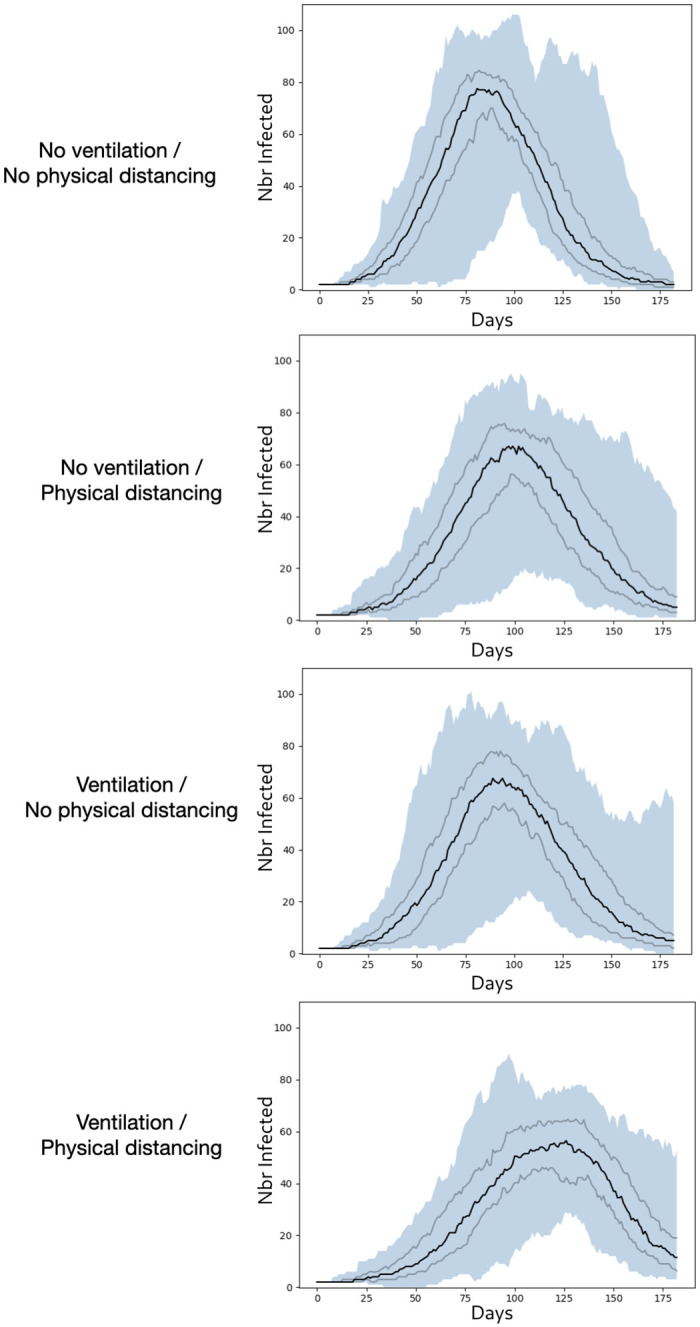
Micro model: Evolution of the number of infected people. The black curve represents the median, the grey curves the first and 3rd quartile respectively, and the blue surface the difference between min and max.

Fig 9 presents the evolution of the number of infected people.

The results show that the application of each individual policy has a minor effect on the spread of the disease, by smoothly flattening the infection curve. However, we can observe a greater response by combining them. In fact, introducing physical distancing into rooms greatly reduces the risk of direct infection. It doesn’t completely eliminate the risk, however, as two people may find themselves at a distance from each other when on the move. It also reduces the risk of infection by objects, as everyone will have their own place in the room, even if in a restaurant, for example, a susceptible person may take the place of an infected person once the latter has left, and thus be infected in turn. Ventilation greatly reduces the risk of airborne infection. However, even with ventilation, the risk of airborne infection is never 0, especially when there are many infected people in the same room. Combining the two effects, and thus considerably reducing all transmission channels, greatly improves the flattening effect, while slowing down the transmission process as the pick of infection occurs from 30 to 50 days after compared to the other’s scenario pick.

### 3.4 COMOKIT-macro

#### 3.4.1 Overview

This model aims at simulating and comparing the application of COVID-19 spread mitigation policies at a scale ranging from a big city to a country. While the COMOKIT-meso and COMOKIT-micro models consider individual people, this model focuses on groups of individuals sharing common characteristics (spatial, physical, social). the transmission of the disease is thus modeled at the scale of individual groups. This model has the same purpose and answers the same questions as the COMOKIT-meso model, but at a much larger scale and with a lower spatial resolution. Thus, contrary to COMOKIT-meso, if it allows to simulate populations composed of millions of inhabitants, it does not allow to test spatial policies that require to have a detailed representation of the space or linked to specific individuals.

To sum up, the model has been designed to be adapted to any scale and in particular regional scales such as a city or a country. The spatial units considered can represent an individual building, a neighborhood, a city, or a region. The simulation step is by default set to 1 hour (but it is possible to take into account a less fine time step). By default, movements from one activity place to another one are not simulated: groups of individuals are always located in an activity place.

**Fig 10:**
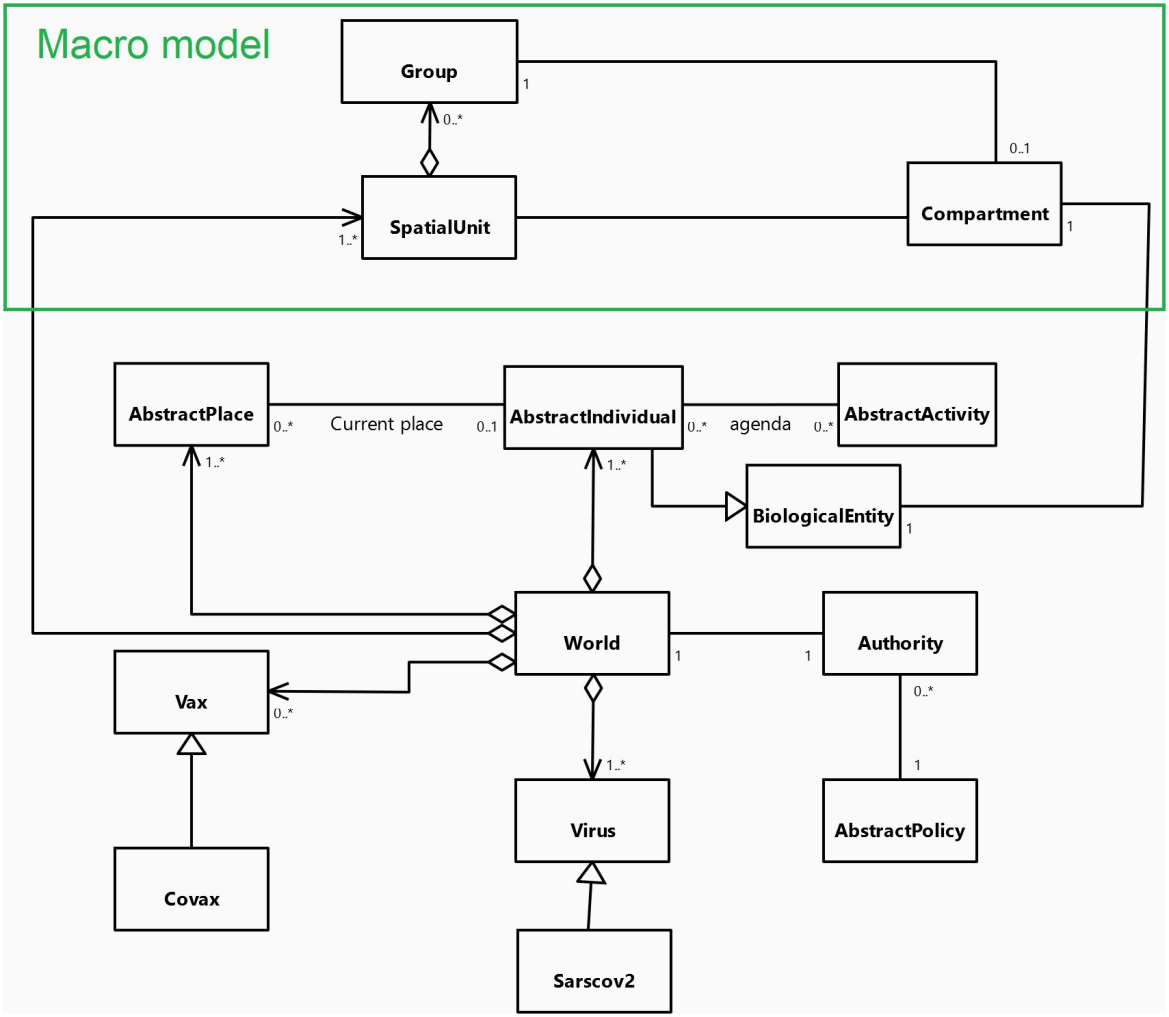
UML class diagram of the COMOKIT Macro entities.

All the agent species are summarized and organized on the UML class diagram presented in Fig 10.

#### 3.4.2 Entities

The core entity of the model is *Group* type of agents: it represents a homogeneous group of individual inhabitants that share similar characteristics with respect to the disease and governance response (incubation time, likelihood of hospitalization, likelihood of wearing a mask, likelihood of following restrictions…). A Group allows to know the number of individuals in the group with each of the epidemiological status (susceptible, latent, pre-symptomatic, symptomatic, asymptomatic…). To take into account the temporal evolution of the disease, the number of individuals in each status is not kept as a simple integer, but as a table, each cell of the table corresponding to one day: e.g. the integer in the second cell of the latent status table represents the number of individuals in the latent status for 2 days.

Another important type of agent is *Compartment*, which is an encapsulation of a Group, which represents a group of inhabitants living in the same *Spatial*_*unit* and which shares the same agenda. The agenda describes, depending on the day of the week and the time of day, the number of people in the *Compartment* who will go to each *Spatial*_*unit*, to perform this or that type of activity in the different types of buildings.

*Spatial*_*unit* agents are spatial entities where the individuals can perform an activity. They are characterized by a set of Compartment agents representing the inhabitants of the area, by a set of building types and for each one by the area that it represents, and finally by a set of *Group* agents representing at the current time step the individuals that are located in the area for each type of building.

Similarly to COMOKIT-meso, the COMOKIT-macro model integrates an *Authority* agent that can allow or not individuals of *Compartment* agents to carry out an activity in a given *Spatial*_*unit* and building type. When a *Compartment* agent asks it for authorization to perform an activity, the *Authority* asks all the policies it has adopted the allowance rate for the *Compartment* to do the given *Activity*.

All the agent species are summarized and organized on the UML diagram presented in Fig 10.

#### 3.4.3 Processes

Similarly to COMOKIT-meso, the dynamics of the model can be summarized by four main dynamics: the epidemic status updating of *Compartment* agents, the activities of the *Compartment* agents, the disease spreading, and the dynamics of policy adoption and application.

At the beginning of each new day, the epidemiological status of the group of each *Compartment* agent is updated. More precisely, for each epidemiological status, the number of individuals in each cell will be transferred to the next table cell, corresponding to the next day. For the number of individuals in the last cell, a vector of transition rates is used to dispatch individuals to the corresponding statuses. As a reminder, all individuals in the same compartment share exactly the same clinical profile (duration of states, severity of illness, immune response, etc.). Thus, only one transition rate from one state to another is used for the compartment. For example, if the transition rate from latent to symptomatic is 0.3 and there are 12 agents in the last cell of the latent table, 4 will be moved to the first cell of the symptomatic table.

The most important dynamic, which is triggered at each simulation step, is the movement of individuals to conduct their activity. More precisely, each *Compartment* agent will calculate according to its agenda and current policies the number of individuals who will go to each *Spatial*_*u*_*nit* agent to conduct their activity in a given type of building. This number will then be managed as a group agent located in the target *Spatial*_*u*_*nit* agent and linked to a building type. The set of these groups will then allow to calculate the number of susceptible individuals that will be infected for each Compartment agent. Unlike the Micro and Meso models, there is no specific *Outside* spatial unit to manage epidemic dynamics outside the studied area. However, modelers are free to define a new specific spatial unit for this purpose.

Finally, the *Authority* agent checks its current *Policy* and tries to apply it (e.g. executing a test campaign for example).

#### 3.4.4 Example of application

In this section, we propose an application of the COMOKIT-macro model to the study of containment policies at the scale of a French department (Alpes-Maritimes, in South-East France) with a population of over one million.

**Fig 11:**
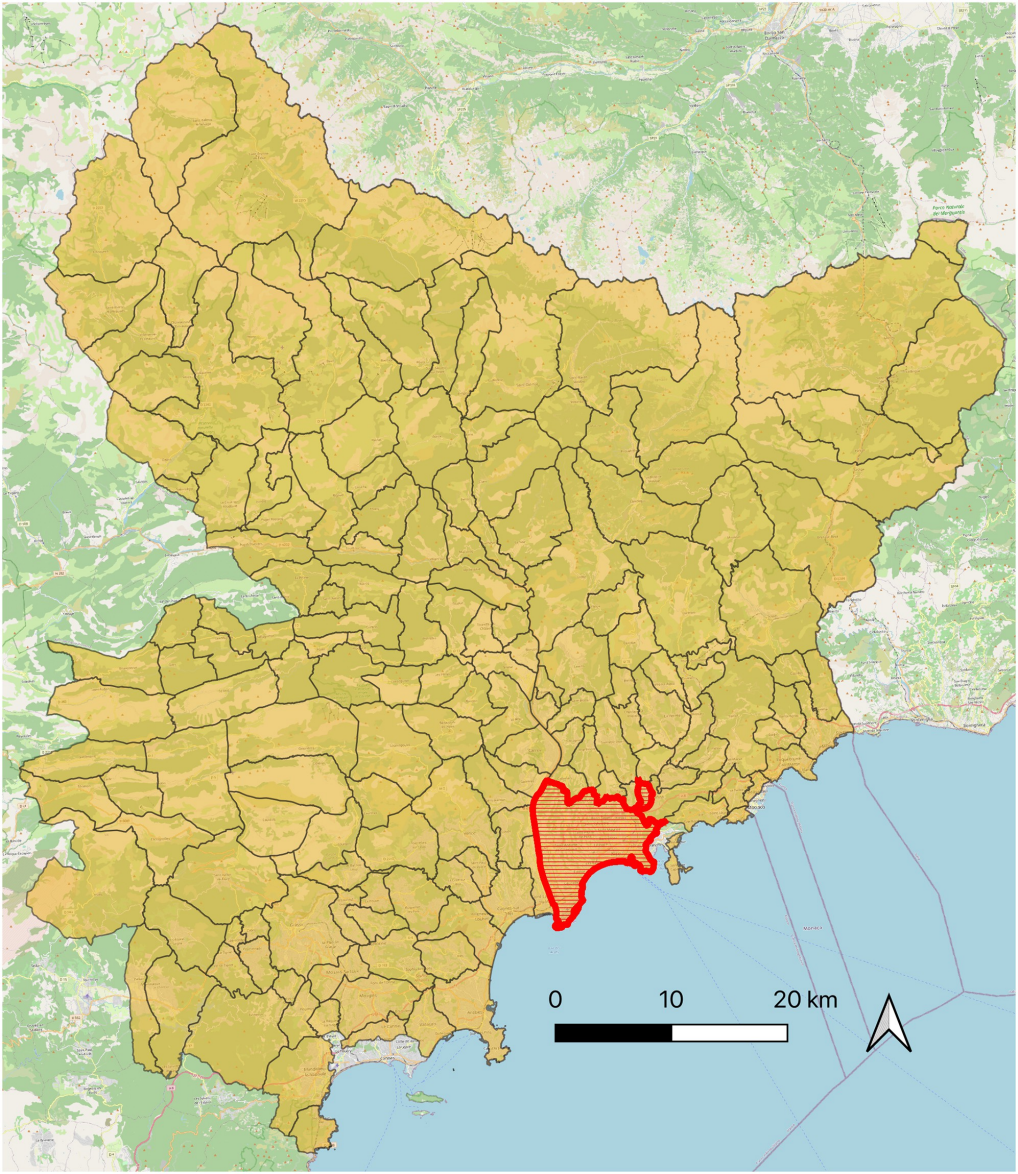
Map of Alpes Maritimes with the different spatial areas considered for the application of the Macro model (data source: OpenStreetMap). The spatial area used for the Meso model experiments is shown in red.

Fig 11 shows a map of the department with its various communes, each commune corresponding to a *Spatial*_*unit* in COMOKIT-macro. By way of comparison, the commune used for the Micro model experiments is shown in red. The population of the area is made up of 1, 097, 410 people for a surface area of 4, 299*km*^2^. To generate the populations we used one of the Gen* generators [15]; Gen* is a synthetic population generator, integrated inside GAMA [16], which uses classical algorithms [17] to generate and spatialize synthetic populations from a variety of data (statistical data, samples, spatial data, etc.). For the generation of agendas, we used the latest household mobility surveys of Alpes-Maritimes to generate synthetic agendas for the different days of the working week. We then used COMOKIT’s built-in generator to reconstruct the weekend agendas. Finally, we aggregated the individuals into groups. In this application, we only used age to constitute these groups: “under 20”, “20 to 44”, “45 to 54”, “55 to 64”, “64 to 74”, “75 to 84” and “over 84”. The reason we chose age is that people’s agendas seem to differ greatly according to their age, and the impact of the disease also depends strongly on people’s age.

In terms of health policy, we consider strict containment (no outside activities allowed and full compliance by inhabitants). Of course, if necessary, COMOKIT would allow us to consider a certain flexibility, whether in terms of tolerance of certain activities or in terms of individuals’ compliance with confinement rules. We have tested different durations: 10 days, 30 days, 60 days, and 90 days, and for each, we compare this policy with a reference scenario in which no policy is applied.

As it is the case for the two other models, the simulation stochasticity has been compensated by carrying out 50 simulations for each value of the parameter.

**Fig 12:**
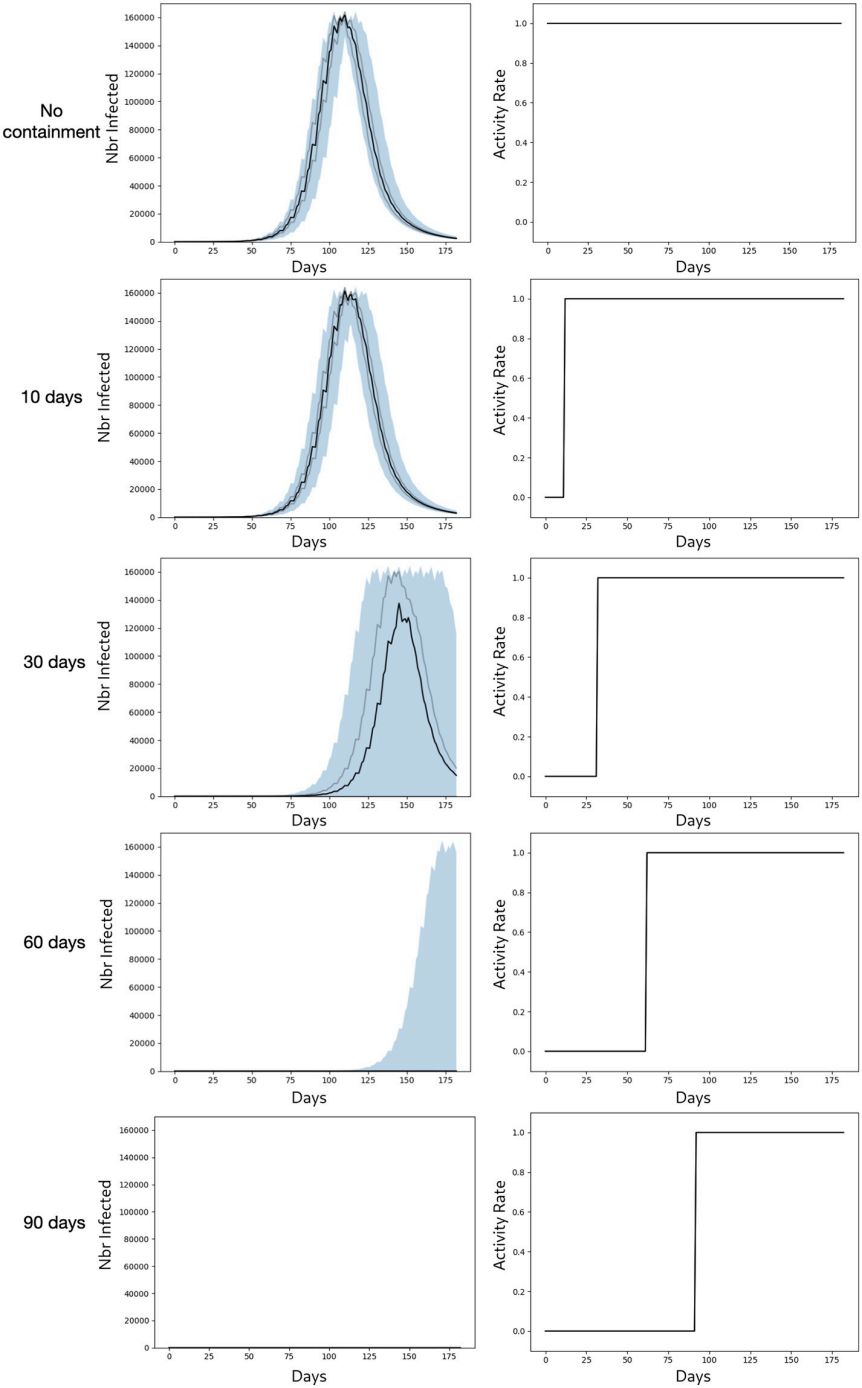
Macro model: Evolution of the number of infected people and of the rate of allowed activities. The black curve represents the median, the grey curves the first and 3rd quartile respectively, and the blue surface the difference between min and max.

Fig 12 shows the evolution of the number of infected individuals and the rate of those who were able to exercise their activity.

The results show that, to be effective, confinement must be sufficiently long. A 10-day confinement only slightly delays the spread of the disease. A 30-day confinement can, in some cases, completely stop the spread of the disease, but in most cases, it only slows it down. At 60 days, it stops the spread of the disease in almost all cases. And after 90 days, the risk of the disease spreading becomes negligible.

## 4 Discussion

One of the great advantages of this new version of COMOKIT is that it offers 3 models working at different scales while sharing a large number of common concepts and abstractions. This feature not only broadens the range of questions that COMOKIT can answer but also makes it possible to envisage integrated multi-level models incorporating different levels of analysis.

Indeed, it is likely that a single model will not be able to answer all the questions raised by the policies to be implemented during a large-scale epidemic, and that not all questions will be relevant at all scales. What’s more, a highly detailed model (such as COMOKIT-micro) requires data that is not always available on a large scale, as well as computing power that can be considerable to take account of its stochasticity. Conversely, a more synthetic model (such as COMOKIT-Macro), by “averaging” individual behaviors, may be more efficient and less data-intensive, but at the same time unable to answer precise questions, such as how to implement a policy on a very local scale. However, beyond the response offered by this range of models to the limitations of a single model, the integration of models makes it possible to tackle questions that simply cannot be tackled in any other way: for example, understanding the global impact (on the scale of a region or country) of a local social distancing policy (on the scale of schools, or companies) requires coupling the two scales of representation, analysis, and experimentation.

The implementation of several different models working in concert is central to the construction of multi-level models capable of embracing different questions. The philosophy behind this type of construction, and the way COMOKIT v2 has been built, is to propose models that are relevant and effective at each scale (individuals, groups, populations) while providing the abstractions and tools to couple these models in such a way that their integration is simple, coherent and enables the initial questions to be answered. In addition, the fact that COMOKIT v2 offers models that are both independent and capable of working together simplifies the modeling task by enabling modelers to focus on one level at a time, and then on their interactions between scales.

Concerning the increasing complexity of models used in public health policy definition, another advantage of having a collection of models that can work independently but share the same abstractions is that they can easily be reused (in part or in whole) in other models. For example, the meso model has already been reused and adapted to several different case studies (e.g., neighborhoods in the city of Nice, France ([18]) or refugee camps [19]). This multi-level architecture also makes it possible to take advantage of certain applications to tackle other case studies: the results obtained at one level (e.g. on the districts of the city of Nice) were thus used to directly define the value of certain parameters and calibrate the model built at the level of the Alpes-Maritimes department.

An important aspect is that COMOKIT v2’s architecture is based on a coherent hierarchy of spatial and temporal representations at micro, meso, and macro levels. This makes it very simple to implement various classic multi-level design architectures, such as those described by [20]: *Zoom*, *Russian Doll* or *Collaboration*. Implementing an architecture such as *Zoom*, dynamically coupling COMOKIT-micro models to a meso or macro implementation, would enable a user to dynamically switch from one scale to another (i.e., to *zoom* in a small-scale model) without jeopardizing the overall dynamics. The *Russian Doll* design pattern would make it possible to observe the evolution of the disease at several levels and scales, enabling even more precise policies to be implemented at the national level. Finally, the *Collaboration* architecture could allow COMOKIT’s framework to be extended or reused in larger models focused on other questions, enabling straightforward integration of disease spread and policy applications.

Whichever coupling pattern is chosen, having common, linked abstractions at all these scales, brought together in the *core* component, in a clear conceptual framework (for example, the *Building* entity of COMOKIT-meso is extended and detailed by the *Room* entity of COMOKIT-micro, both being specific types of *AbstractPlace* entities of the *core* component), represents a considerable advantage for not only using but also extending COMOKIT to other diseases or uses.

## 5 Conclusion and perspectives

In this article, we present version 2 of COMOKIT, a comprehensive toolbox for modeling and simulating the impact of infectious disease management policies at different scales. COMOKIT integrates 3 abstract models to address issues ranging from the management of contamination risks inside buildings to the evaluation of containment or vaccination policies at the scale of a city or even a country. To illustrate its use, we describe 3 applications of this toolbox, each with its own issues and characteristics: COMOKIT-micro, capable of taking into account the precise location of individuals in buildings and the characteristics of the rooms they are in, illustrates the value of a model for studying the effects of physical distancing in conjunction with room ventilation; COMOKIT-meso, capable of taking into account the spatial organization of buildings within neighborhoods, as well as the daily agenda of each individual, is used to understand the spatial reach of localized containment policies in a neighborhood; finally, COMOKIT-macro, thanks to its ability to represent the behaviors and dynamics of groups of individuals, is used to better understand the impact of containment measures on the scale of a French region (over a million inhabitants).

Although the first applications of COMOKIT concern COVID-19, the toolbox has been designed to be generic and adaptable to any type of airborne contagious disease. Indeed, the only task in adapting COMOKIT to these diseases will be to define new *Virus* agents and adapt the parameters of the disease in question. This is a direction we have already begun to explore with the seasonal influenza viruses. To go even further, and to exploit the very great flexibility and versatility of the agent-based approaches, we plan to enhance COMOKIT to include the possibility of representing vector-borne diseases such as dengue fever. To achieve this, COMOKIT’s *core* will have to be extended with the definition of vectors and hosts, and their dynamics, not only epidemiological but also behavioral, while preserving the ease of use of existing abstractions.

While COMOKIT has proved in several applications to be a great help in thinking about the policies to be put in place to deal with an epidemic, it is nevertheless based, like any model, on a certain number of simplifying assumptions, both on epidemiological aspects and on individual behaviors. If it is possible to improve these aspects in COMOKIT by allowing finer parameterization of the model or new abstractions, another interesting avenue is to couple COMOKIT with other models enabling, for example, better representation of the dynamics of infection (e.g. environmental transmission, by animals…) or the mobility of individuals (e.g. specificity of modes of transport such as air or rail). The current evolution of the GAMA platform towards greater interoperability with platforms such as MATSim [21] should enable this type of synergy to be explored in the near future.

Still in this perspective, and as mentioned in the discussion, one of the advantages of COMOKIT V2 is the definition of common abstractions at 3 different scales, making it easy to switch from one representation to another depending on the question. For the time being, this is achieved by defining different models. One prospect we are working on is to propose an even more integrated approach to facilitate these scale transitions, by offering the possibility of simultaneously having several levels of representation and analysis in the same integrated model. For example, a simulation on the scale of a neighborhood (COMOKIT-meso model) gives the possibility to represent and simulate precisely what happens inside a particularly critical building (a hospital) with the COMOKIT-micro model. Or, in the case of hygiene rules imposed on a school scale (COMOKIT-micro), be able to represent and simulate how individual variations influence the overall evolution of disease on a regional scale (COMOKIT-macro).

The modeling of socio-environmental phenomena is increasingly moving towards the construction of complex integrated models, capable of taking into account individual behavior, global dynamics, and their intricate interactions. This evolution subjects modelers to constraints that software engineers have long been familiar with (around the definition of design frameworks or coupling strategies), reinforced by the interdisciplinarity that characterizes the major questions posed by our societies. In the specific case of epidemiology, we are confident that, thanks to its architecture and openness, COMOKIT and its numerous descendants will enable us to tackle questions that are still too difficult to represent in conventional modeling, and thus provide better input for public decision-making.
